# $N^{3/4}$ Law in the Cubic Lattice

**DOI:** 10.1007/s10955-019-02350-z

**Published:** 2019-07-24

**Authors:** Edoardo Mainini, Paolo Piovano, Bernd Schmidt, Ulisse Stefanelli

**Affiliations:** 1 Dipartimento di Ingegneria meccanica, energetica, gestionale e dei trasporti, Università degli studi di Genova, Via all’Opera Pia 15, 16145 Genoa, Italy; 2 Faculty of Mathematics, University of Vienna, Oskar-Morgenstern-Platz 1, 1090 Vienna, Austria; 3 Institute of Mathematics, University of Augsburg, Universitätsstr. 14, 86159 Augsburg, Germany; 4 Istituto di Matematica Applicata e Tecnologie Informatiche E. Magenes - CNR, via Ferrata 1, 27100 Pavia, Italy

**Keywords:** Wulff shape, $N^{3/4}$ law, Cubic lattice, Fluctuations, Edge perimeter, 82D25

## Abstract

We investigate the Edge-Isoperimetric Problem (EIP) for sets with *n* elements of the cubic lattice by emphasizing its relation with the emergence of the Wulff shape in the crystallization problem. Minimizers $M_n$ of the edge perimeter are shown to deviate from a corresponding cubic Wulff configuration with respect to their symmetric difference by at most $\mathrm{O}(n^{3/4})$ elements. The exponent 3 / 4 is optimal. This extends to the cubic lattice analogous results that have already been established for the triangular, the hexagonal, and the square lattice in two space dimensions.

## Introduction

In this contribution we consider the *Edge-Isoperimetric Problem* (EIP) in

$$\begin{aligned} \mathbb {Z}^3:=\{k_1 \varvec{e}_1+k_2\varvec{e}_2+ k_3 \varvec{e}_3\,\,:\quad k_i\in \mathbb {Z}\text { for }i=1,2,3\} \end{aligned}$$

where $\varvec{e}_1:=\left( 1,0,0\right)$, $\varvec{e}_2:=(0,1,0)$ and $\varvec{e}_3:=(0,0,1)$. For any set $C_n$ made of *n* elements of $\mathbb {Z}^3$, we denote by $\Theta (C_n)$ the *edge boundary* of $C_n$, i.e.,

1 $$\begin{aligned} \Theta (C_n):=\big \{ (x,y)\in \mathbb {Z}^3\times \mathbb {Z}^3 \ : \ |x-y|=1,\, x\in C_n\text { and }y\in \mathbb {Z}^3{\setminus } C_n \big \} \end{aligned}$$

and we refer to its cardinality $\#\Theta (C_n)$ as the *edge perimeter* of $C_n$. Notice that the edge perimeter of a set $C_n$ coincides with the surface area of the union of the closure of the Voronoi cells related to $C_n$, i.e.,

$$\begin{aligned} \#\Theta (C_n):= \mathcal {H}^2(\partial \{x\in \mathbb {R}^3\, :\, \text {dist}(x,C_n)\le \text {dist}(x,\mathbb {Z}^3 {\setminus } C_n)\}), \end{aligned}$$

where $\mathcal {H}^2$ denotes the two-dimensional Hausdorff measure in $\mathbb {R}^3$. Given the family $\mathcal {C}_n$ of all sets $C_n \subset \mathbb {Z}^3$ with *n* elements, the Edge-Isoperimetric Problem over $\mathcal {C}_n$ consists in considering the minimum problem

2 $$\begin{aligned} \theta _n:=\min _{C_n\in \mathcal {C}_n} \#\Theta (C_n), \end{aligned}$$

which we denote by EIP$_n$, and in characterizing the EIP$_n$ solutions. The EIP is a classical combinatorial problem and a review on the results in Combinatorics can be found in [2, 12]. Beyond its relevance in pure combinatorics, the EIP (and corresponding problems for similar notions of perimeter) plays a decisive role in a number of applied problems, ranging from *machine learning* (see [23] and references therein) to the *Crystallization Problem* (CP). We refer the reader to [9] for the relation between the EIP in the triangular lattice and the CP with respect to a two-body interatomic energy characterized by the *sticky-disc* interaction potential (see [13, 19] for more details).

The minimum problem (2) relates also to the Ising model for ferromagnetic materials that characterizes the magnetism of a bulk material as the combined effect of magnetic dipole moments of the many atomic spins within the material [10]. Such magnetic dipole moments are generally considered to be arranged on each site of a fixed lattice and assumed to be in two states, either $+1$ or $-1$. The connection with (2) resides on the fact that the Hamiltonian of the Ising model can be expressed under certain conditions with respect to the edge perimeter of the plus phase, i.e., the set of $+1$ spins, as described in [10].

Our main objective is to prove that any minimizer of the EIP$_n$ –after a suitable translation– differs from a fixed *cubic configuration*

3 $$\begin{aligned} W_n:= [0,\ell _n]^3\cap \mathbb {Z}^3 \quad \text{ with }\quad \ell _n:=\lfloor \root 3 \of {n}\rfloor . \end{aligned}$$

(with respect to the cardinality of their symmetric difference) by at most

4 $$\begin{aligned} K\, n^{3/4} + \mathrm{o}(n^{3/4}) \end{aligned}$$

elements of $\mathbb {Z}^3$ for some universal positive constant $K>0$ (see Theorem 1.1), and to show that this estimate is sharp for infinitely many *n*. In particular, the exponent 3 / 4 of the leading term cannot be lowered in general. The right scaling intuition comes from the lower bound construction (see Sect. 4), that allows to see how much one can ‘dig’ a ground state, still keeping minimality. In the following we refer to the cubic configuration $W_n$ as the *Wulff shape* because of the analogy to the crystallization problem and the Ising model. For results in Statistical Mechanics related to the derivation in the scaling limit of the Wulff shape in the context of the low-temperature two-dimensional Ising model we refer to [10] (see also [14, 15, 21, 22]), and to [4, 7] for analogous results in three dimensions. Results in Combinatorics can be found in [6], where some of the minimizers of the edge perimeter in the family of polyominoes are characterized.

We first show that (4) is an *upper bound* for every minimizer of EIP$_n$.

### Theorem 1.1

(Upper bound) There exists a constant $K_1>0$ independent of *n* such that

5 $$\begin{aligned} \min _{a \in \mathbb {Z}^3} \#(M_n\triangle (a + W_n) ) \le K_1 n^{3/4} + \mathrm{o}(n^{3/4}) \end{aligned}$$

for every $n\in \mathbb {N}$ and every minimizer $M_n$ of the EIP$_n$.

Our second result shows that the exponent 3 / 4 in (4) is optimal.

### Theorem 1.2

(Lower bound) There exists a sequence of minimizers $M_{n_i}$ with a diverging number $n_i\in \mathbb {N}$ of particles such that

6 $$\begin{aligned} \min _{a \in \mathbb {Z}^3} \#(M_{n_i}\triangle (a + W_{n_i}) ) \ge K_2 {n_i}^{3/4} + \mathrm{o}(n_i^{3/4}) \end{aligned}$$

for some constant $K_2>0$ (not depending on $n_i$).

We will prove Theorem 1.1 in Sect. 3 and Theorem 1.2 in Sect. 4. By setting $K := \limsup _{n\rightarrow \infty } n^{-3/4} \max _{M_n} \min _{a \in \mathbb {Z}^3} \#(M_n\triangle (a + W_n) )$, where the maximum is taken among all configurations $M_n$ that are EIP$_n$ minimizers, by Theorems 1.1 and 1.2 we have $K\in (0,+\infty )$. We see that it is possible to choose $K_1 = K_2 = K$ in (5) and (6).

These results for $\mathbb {Z}^3$ are the first ones related to fluctuations of minimizers in three dimensions. Analogous results in two dimensions have been established in [9, 20] for the triangular lattice (see also [1]), in [17, 18] for the square lattice, and finally in [8] for the hexagonal lattice. The methods in these contributions are based on rearrangement techniques [20] and on the isoperimetric characterization of minimizers (with respect to suitable notions of perimeter *P* and area *A* of configurations) which also allows one to find the optimal constants for relations of the type of (5) (see [8, 9]). Quite surprisingly, the same exponent 3 / 4 is optimal in all of the two-dimensional cases considered (triangular, square, hexagonal) and in $\mathbb {Z}^3$. This is however not a general fact, for other exponents are to be expected in $\mathbb {Z}^d$ for $d\ge 4$, see the end of Sect. 4.

The strategy of the proofs is mainly based on generalizing to three dimensions the rearrangement techniques used in [17, 20], and using the fine characterization of the edge perimeter for minimizers of the EIP in two dimensions obtained in [17]. The analogue in three dimensions of the two-dimensional passing to a *normalized ground state* as in [20] and the *rectangularization* employed in [17] is here the *cuboidification* (see Definition 2.2), which is quite more involved and allows to pass from any minimizer to a *quasicubic* minimizer (see Definition 2.4). However, to obtain the upper bound further transformations are needed in particular to prove the relation (25) between the largest dimension of a minimizer and the side of the base of the corresponding cuboidification. To that end, we show how quasicubes can be rearranged to be close to a cube with a “hole” near one of the corners by moving many atomic layers at once, the (considerably more elementary) two dimensional version of which was crucial in [20]. We also make use of the edge-perimeter characterization for the solutions of two dimensional EIP [17]. The lower bound relies on a refinement of the argument for the two-dimensional case, which we establish in Lemma 4.1. As a by product of this, we prove the sharpness of the $n^{3/4}$ upper bound also for the square lattice, which was not addressed in [17].

## Mathematical Setting

In this section we introduce the main definitions and notations used throughout the paper.

We first recall a useful characterization of EIP$_n$ minimizers that we shall often exploit. The number of (unit) *bonds* of a configuration $C_n\in \mathcal {C}_n$ is

$$\begin{aligned} b(C_n):=\frac{1}{2} \,\#\big \{(x,y)\in C_n \times C_n: |x-y|=1\big \}. \end{aligned}$$

Then the elementary relation $\#\Theta (C_n)+2b(C_n)=6n$ shows that $C_n$ is a minimizer for the EIP$_n$ if and only if it maximizes the number of unit bonds.

We also introduce the 2-dimensional analogon of (2) which we denote here as EIP$^2_d$ for $d\in \mathbb {N}$, i.e.,

7 $$\begin{aligned} \eta _d:=\min _{E_d\in \mathcal {C}^{2}_d} \#\Theta _{2}(E_d), \end{aligned}$$

where $\mathcal {C}^{2}_d$ is the family of subsets of the square lattice $\mathbb {Z}^2$ with *d* elements and

8 $$\begin{aligned} \Theta _2(E_d):=\left\{ (x,y)\in \mathbb {Z}^2\times \mathbb {Z}^2 \ : \ |x-y|=1,\, x\in E_d\text { and }y\in \mathbb {Z}^2{\setminus } E_d \right\} . \end{aligned}$$

We recall from [17] that $E_d$ solves (7) if and only if the number of unit bonds of $E_d$ (i.e., $\tfrac{1}{2} \,\#\{(x,y)\in E_d\times E_d: |x-y|=1\}$) is equal to $\lfloor 2d-2\sqrt{d}\rfloor$. This is equivalent to $\#\Theta _{2}(E_d) = 4d - 2\lfloor 2d-2\sqrt{d}\rfloor$, i.e., to

9 $$\begin{aligned} \#\Theta _{2}(E_d) = 2\lceil 2\sqrt{d} \rceil . \end{aligned}$$

We also recall from [17] that for any $d\in \mathbb {N}$ there exists a minimizer $D_d$ of EIP$^2_d$ of the type

10 $$\begin{aligned} D_d:=R(s,s')\cup L_e \end{aligned}$$

for some $s,s'\in \mathbb {N}$ and $e\in \mathbb {N}\cup \{0\}$ such that $s'\in \{s,s+1\}$, $s\cdot s'+e=d$, and $e< s'$, where

11 $$\begin{aligned} R(s,s'):=\mathbb {Z}^2\cap \left( [1,s]\times [1,s']\right) \end{aligned}$$

and

12 $$\begin{aligned} L_e := {\left\{ \begin{array}{ll}\mathbb {Z}^2\cap \left( (0,e]\times \{s'+1\} \right) \quad &{}\text {if }s'=s,\\ \mathbb {Z}^2\cap \left( \{s+1\}\times (0,e] \right) \quad &{}\text {if }s'=s+1. \end{array}\right. } \end{aligned}$$

**Fig. 1:**
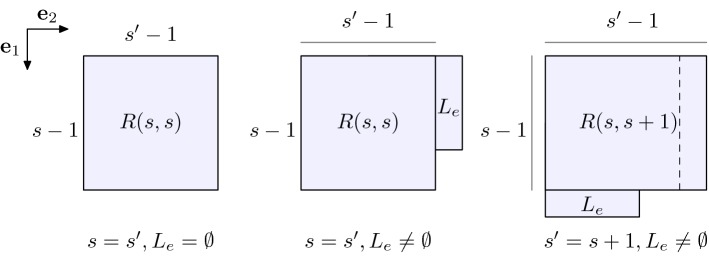
The *daisies*$D_d$, *d* increases from left to right

Notice that if $e=0$, then $L_e=\emptyset$, see Fig. 1. As already done in [17], we refer to these two-dimensional minimizers as *daisies*. In fact, the name originates from the hexagonal case [16], where the analogous construction gives rise to hexagonal configurations of hexagons, resembling a flower. Moreover, we term the integers $s-1$ (resp. $s'-1$) as the minimal (resp. maximal) *side length* of the rectangle (11) of the daisy.

Let us also introduce the notion of *minimal rectangle* associated to a 2-dimensional configuration $C_n$ in $\mathbb {Z}^3$.

### Definition 2.1

Given a configuration

$$\begin{aligned} C_n\subset \{k_1 \varvec{e}_1+k_2\varvec{e}_2+ z \varvec{e}_3\,\,:\quad k_i\in \mathbb {Z}\text { for }i=1,2\} \end{aligned}$$

for some $z\in \mathbb {Z}$, we denote by $R(C_n)$ the closure of the *minimal rectangle* containing $C_n$, i.e., the minimal rectangle *R* with respect to set inclusion in

$$\begin{aligned} \mathbb {R}^2_z:=\{(z_1,z_2,z_3)\in \mathbb {R}^3\,:\, z_3=z\} \end{aligned}$$

with sides parallel to $\varvec{e}_i$ for $i=1,2$, and that satisfies $C_n\subset R$.

Moving ahead to 3-dimensional configurations in $\mathbb {Z}^3$ we introduce here a discrete rearrangement procedure, which we call *cuboidification*. Notice that the cuboidification is the 3-dimensional analogue of the 2-dimensional rearrangement introduced in [17] and denoted *rectangularization* (see also [5, 11, 12]), even though here we define such rearrangement only for EIP$_n$ minimizers and not for a general configuration. To this end, let us introduce for every $z\in \mathbb {Z}$ the notion of *z*-levels of a configuration $C_n\subset \mathbb {Z}^3$ in the direction $i=1,2,3$, i.e., the 2-dimensional configurations defined by

$$\begin{aligned} C_n(z, \cdot ,\cdot )&:=C_n\cap \{z \varvec{e}_1+k_2\varvec{e}_2+ k_3 \varvec{e}_3\,\,:\quad k_i\in \mathbb {Z}\text { for }i=2,3\}, \nonumber \\ C_n(\cdot , z,\cdot )&:=C_n\cap \{k_1 \varvec{e}_1+z\varvec{e}_2+ k_3 \varvec{e}_3\,\,:\quad k_i\in \mathbb {Z}\text { for }i=1,3\}, \quad \text {and}\\ C_n(\cdot ,\cdot , z)&:=C_n\cap \{k_1 \varvec{e}_1+k_2\varvec{e}_2+ z \varvec{e}_3\,\,:\quad k_i\in \mathbb {Z}\text { for }i=1,2\}, \end{aligned}$$

respectively. In the following, we also denote by $Q(a_1,a_2,a_3)$ for some $a_i\in \mathbb {N}$ the closed cuboid

$$\begin{aligned} Q(a_1,a_2,a_3):=[1,a_1] \times [1,a_2]\times [1,a_3]. \end{aligned}$$

Furthermore, we define the *3-vacancies* of a configuration $C_n\subset \mathbb {Z}^3$ as the elements of $\mathbb {Z}^3{\setminus } C_n$ that would activate three bonds if added to $C_n$, i.e., those elements of $\mathbb {Z}^3{\setminus } C_n$ which have a distance 1 to exactly three different elements of $C_n$.

**Fig. 2:**
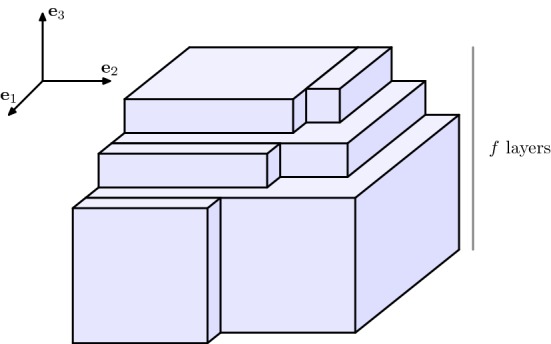
Configuration $M'_n$. A caveat: in favor of illustrative clarity, proportions in this and the following figures do not correspond to the actual ones of a ground state

**Fig. 3:**
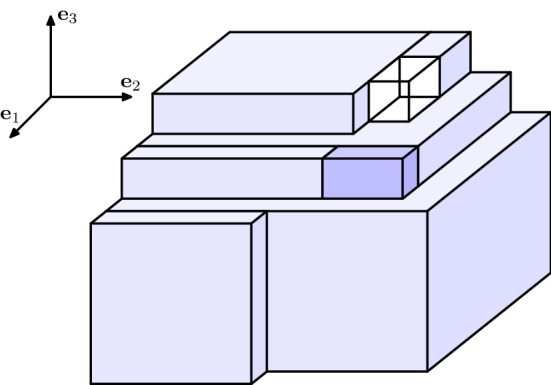
Configuration $$M''_n$$

### Definition 2.2

We define the *cuboidification*$\mathcal {Q}(M_n)$ (in the direction $\varvec{e}_3$) of a minimizer $M_n$ of the EIP$_n$ as the configuration resulting from rearranging the particles of $M_n$ according to the following three steps.

**Fig. 4:**
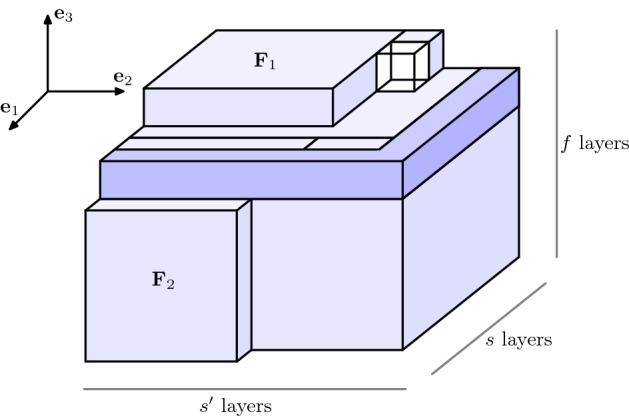
Configuration $$M'''_n$$

(i) For every $z\in \mathbb {Z}$, let $d_z:=\#M_n(\cdot ,\cdot , z)$ and consider the 2-dimensional daisy $D_{d_z}$ (which has been defined in (10)), order the elements of the family $( D_{d_z} )_{d_z\ne 0}$ decreasingly with respect to their cardinality, say $\displaystyle (D^{(k)})_{k=1,\dots ,f}$ with $f:= \#\{ z \in \mathbb {Z}\,:\,d_z\ne 0 \}$, and consider the configuration $M'_n$ characterized by
  $$\begin{aligned} M'_n(\cdot ,\cdot ,k)=D^{(k)}+k\varvec{e}_3\end{aligned}$$
  for $k=\{1,\ldots , f\}$ and $M_n'(\cdot ,\cdot ,k)=\emptyset$ if $k\notin \{1,\ldots ,f\}$, see Fig. 2.
  By (10) there exist $s,s'\in \mathbb {N}$ and $e\in \mathbb {N}\cup \{0\}$ with $s\cdot s'+e=d$, $s'\in \{s,s+1\}$, and $e< s'$, such that $D^{(1)}= R(s,s') \cup L_e$ for $R(s,s')$ and $L_e$ defined as in (11) and (12), respectively.
  It is clear that $M_n'$ is still an EIP$_n$ minimizer. Also, if $f\le 2$ the cuboidification algorithm ends here. Otherwise, we proceed to the next steps.
(ii) Consecutively move the elements from $M'_n(\cdot ,\cdot ,f)$ with at most 3 bonds (there is always at least one of them) to fill the 3-vacancies in $M'_n{\setminus } M'_n(\cdot ,\cdot ,f)$. This allows to obtain a configuration $M''_n$ whose levels $M''_n(\cdot ,\cdot ,k)$ for $k=2,\dots ,f-1$ are rectangles (with possibly the extra segment $L_e+k\varvec{e}_3$), i.e.,
  13 $$\begin{aligned} M''_n(\cdot ,\cdot ,k){\setminus }(L_e+k\varvec{e}_3)=R(a_k,b_k) \end{aligned}$$
  for some $a_k$, $b_k\in \mathbb {N}\cup \{0\}$ which are decreasing in *k*, see Fig. 3. We also notice that the $(z=1)$-level remains unchanged, i.e.,
  $$\begin{aligned} M''_n(\cdot ,\cdot ,1)=M'_n(\cdot ,\cdot ,1)=D^{(1)}+\varvec{e}_3, \end{aligned}$$
  and we assume, without loss of generality, that $M''_n(\cdot ,\cdot ,f)$ is a daisy. We stress that three-vacancies, if any, are filled one by one, first at the level $z=2$, and then at the levels $z=3,\ldots f-1$ (in this order), in such a way that all the *z*-levels up to $f-1$ are still daisies. Moreover, each of these daisies is either coinciding with $M''_n(\cdot ,\cdot , 1)$, or it is a rectangle: that is, if (13) holds and $a_k<s$ or $b_k<s'$, then actually $M_n''(\cdot ,\cdot ,k)=R(a_k,b_k)$. As the three-vacancies are filled with atoms that present at most 3 bonds, the total number of bonds does not decrease and hence, $M''_n$ is also an EIP$_n$ minimizer.
(iii) We now construct a configuration $M'''_n$ by iteratively performing the following procedure $\mathcal {P}_k$ for $k=2,\dots ,f-1$. The procedure $\mathcal {P}_k$ consists of performing the following two substeps:
  1. If $a_k= s$ we directly pass to Substep 2). If instead $a_k< s$, then we move an entire external edge from the *f*-level (an *f*-level edge smaller or equal to $b_k$ exists by Step (ii)), attach it at the *k*-level so that each of its atoms is bonded both to an atom that was already at the *k*-level and to one atom at the $(k-1)$-level, and, if any 3-vacancies at the *k*-level appeared, then we repeat Step (ii) in order to fill them. This can be performed so that the *k*-level of the obtained configuration is $R(a_k+1,b_k)$. By iterating this substep $a=s-a_k$ times, the *k*-level of the resulting configuration is $R(s,b_k)$.
  2. If $b_k= s'$, then the procedure $\mathcal {P}_k$ is finished. If instead $b_k< s'$, then we move an entire external edge from the *f*-level, attach it at the *k*-level so that each of its atoms is bonded both to an atom that was already at the *k*-level and to one atom at the $(k-1)$-level, and possibly remove any 3-vacancies by repeating Step (ii). We then iterate this substep $b=s'-b_k$ times, so that the *k*-level of the final configuration is $R(s,s')$.
  For each $k=2,\ldots , f-1$, the output of this step is a *k*-level of the form $R(s,s')$, plus possibly an extra-line $L_e+k\varvec{e}_3$ (which can be there only if $a_k=s$ and $b_k=s'$, that is, only if the procedure $\mathcal {P}_k$ is empty), see Fig. 4. We notice that, if we denote by $\mathcal {P}_k(M''_n)$ the configuration obtained by iteratively performing $\mathcal {P}_i$ for $i=2,\dots ,k$, we have that
  $$\begin{aligned} \mathcal {P}_k(M''_n)(\cdot ,\cdot ,i){\setminus }(L_e+i\varvec{e}_3)=R(s,s') \end{aligned}$$
  for every $i=2,\dots ,k$, and that
  $$\begin{aligned} M'''_n:=\mathcal {P}_{f-1}(M''_n)=(\mathbb {Z}^3\cap Q(s,s',f-1))\cup F_1\cup F_2 \end{aligned}$$
  where $F_1:=M'''_n(\cdot ,\cdot ,f)$ is rearranged as a daisy and
  $$\begin{aligned} F_2:={\left\{ \begin{array}{ll}M'''_n(\cdot ,s+1,\cdot )\quad \text {if }s'=s,\\ M'''_n(s+1,\cdot ,\cdot )\quad \text {if }s'=s+1. \end{array}\right. } \end{aligned}$$
  We notice that $F_2{\setminus } F_1$ is a rectangle $R(e,s'')$ for some $e\in \{0,\ldots , s'-1\}$, $s''\in \{1,\ldots f-1\}$ and that, if $e=0$, then $F_2=\emptyset$. Without loss of generality we assume that $F_2=M'''_n(s+1,\cdot ,\cdot )$, as we can move the whole $F_2$ (hence also the extra-line of the daisy $F_1$, if such line is contained in $F_2$) on that side of $Q(s,s',f)$ since $s'\ge s$.

The configuration $\mathcal {Q}(M_n):=M_n'''$ is still an EIP$_n$ minimizer and it is the output of the cuboidification.

### Remark 2.3

We stress that the recursive application of Steps (ii) and (iii) in the above definition can never exhaust the upper face $M_n'(\cdot ,\cdot ,f)$ nor break its minimality for the two-dimensional EIP before a configuration of the form of $M_n'''$ is created, otherwise $M_n$ would not be a minimizer of the EIP$_n$.

We conclude this section with two more definitions.

### Definition 2.4

We say that a configuration $C_n$ is *quasicubic*, or a *quasicube*, if there exist $s,s',s_3\in \mathbb {N}$ with $s'\in \{s,s+1\}$ such that (up to translation, relabeling, and reorienting the coordinate axes)

$$\begin{aligned} C_n=\left( \mathbb {Z}^3\cap Q(s,s',s_3-1)\right) \cup F^1_{d_1}\cup F^2_{d_2} \end{aligned}$$

where $F^i_{d_i}$, $i=1,2$, are configurations with cardinality $d_i:=\#F^i_{d_i}$ such that

$$\begin{aligned} F^1_{d_1}\subset \mathbb {Z}^3\cap ([1, s+1]\times [1,s']\times \{s_3\}) \end{aligned}$$

and

$$\begin{aligned} F^2_{d_2}\subset \mathbb {Z}^3\cap (\{s+1\}\times [1,s'-1]\times [1,s_3]). \end{aligned}$$

We observe that by Definition 2.2 the cuboidification $\mathcal {Q}(M_n)$ of an EIP$_n$ minimizer $M_n$ is a quasicube with $s_3=\#\{z\, :\, M_n(\cdot ,\cdot , z)\ne \emptyset \}$ and $s-1$ the smallest side length of the rectangle $[1,s]\times [1,s']$ of the daisy with $\max _z \#M_n(\cdot ,\cdot , z)$ elements. Notice also that similar rearrangement techniques to the cuboidification introduced in Definition 2.2 are employed in [6] to prove that a subclass of the quasicubic configurations of Definition 2.4 have minimal surface area among polyominoes. Finally, we define the minimal cuboid of a configuration $C_n\subset \mathbb {Z}^3$.

### Definition 2.5

Given a configuration $C_n\subset \mathbb {Z}^3$ we denote by $Q(C_n)$ the closure of the *minimal rectangular cuboid* containing $C_n$, i.e., the smallest rectangular cuboid *Q* with respect to set inclusion in $\mathbb {R}^3$ that has sides parallel to $\varvec{e}_i$ for $i=1,2,3$, and such that $C_n\subset Q$.

## Upper Bound: Proof of Theorem 1.1

We exploit the cuboidification algorithm from Sect. 2 to obtain the proof of our first main result.

### Proof of Theorem 1.1

Fix $n\in \mathbb {N}$ and let $M_n$ be a minimizer of EIP$_n$. In the following, without loss of generality (up to a translation and rotation of the coordinate system), we assume that

14 $$\begin{aligned} Q(M_n)=Q(\ell _1+1,\ell _2+1,\ell _3+1) \end{aligned}$$

for some $\ell _1,\ell _2,\ell _3\in \mathbb {N}\cup \{0\}$ with

15 $$\begin{aligned} 0\le \ell _1\le \ell _2 \le \ell _3, \end{aligned}$$

where $Q(M_n)$ is the minimal cuboid of $M_n$ (see Definition 2.5) and $\ell _i$, $i=1,2,3$ its side lengths. Notice also that $Q(W_n)=Q(\ell _n+1,\ell _n+1,\ell _n+1)-(1,1,1)$ for $\ell _n$ defined in (3).

We now claim that there exists a constant $K>0$ (which does not depend on *n* and $M_n$) such that

16 $$\begin{aligned} \displaystyle \max _{i=1,2,3} |\ell _i-\ell _n|\le K n^{1/12} +\mathrm{o} (n^{1/12}). \end{aligned}$$

Once this is proved, Theorem 1.1 follows since $\ell _n=n^{1/3} +\mathrm{O}(1)$ by (3) and hence each external face of $Q(\ell _1+1,\ell _2+1,\ell _3+1)$ intersected with $\mathbb {Z}^3$ has cardinality $n^{2/3}+\mathrm{o}(n^{2/3})$. Thus we obtain

$$\begin{aligned} \min _{a \in \mathbb {Z}^3} \#(Q(M_n)\triangle (a + W_n) ) \le 3K n^{3/4} + \mathrm{o}(n^{3/4}). \end{aligned}$$

By (16) we have

$$\begin{aligned} \#( Q(M_n) {\setminus } M_n )&= (\ell _1 + 1) (\ell _2 + 1) (\ell _3 + 1) - n \\&\le (n^{1/3} + K n^{1/12} + \mathrm{O}(1))^3 - n = 3K n^{3/4} + \mathrm{o}(n^{3/4}), \end{aligned}$$

and so (5) follows.

In order to prove (16) we proceed in 5 steps.

### Step 1

In this step we show that by rearranging the elements of $M_n$ we can construct another minimizer $\overline{M}_n$ of the EIP$_n$ that is quasicubic, i.e., there exists $\ell ,\ell ',\ell _3 \in \mathbb {N}\cup \{0\}$ with $0\le \ell \le \ell _3$, $\ell '\in \{\ell ,\ell +1\}$, and configurations $F^1_{d_1}$ and $F^2_{d_2}$ as in Definition 2.4 such that

17 $$\begin{aligned} \overline{M}_n=\left( \mathbb {Z}^3\cap Q(\ell +1,\ell '+1,\ell _3)\right) \cup F^1_{d_1}\cup F^2_{d_2}. \end{aligned}$$

This assertion follows by choosing $\overline{M}_n=\mathcal {Q}(M_n)$ and by observing that (17) is satisfied with $\ell$ being the smallest side length of the rectangle of the daisy $D_{m}$ (see (10)) where *m* is the maximal cardinality of the *z*-levels $M_n(\cdot ,\cdot , z)$ of $M_n$ for $z=1,\dots ,\ell _3+1$, i.e.,

$$\begin{aligned} m:=\max _{z=1,\dots ,\ell _3+1} \#M_n(\cdot ,\cdot , z). \end{aligned}$$

We notice that

18 $$\begin{aligned} \ell _3\ge \ell . \end{aligned}$$

Indeed, by (14) we have $m\le (\ell _1+1)(\ell _2+1)$. On the other hand, by the definition of daisy, since $\ell$ is the minimal side length of the rectangle of the daisy $D_m$, it clearly satisfies $\ell +1\le \sqrt{m}$. Therefore

19 $$\begin{aligned} \ell +1\le \sqrt{(\ell _1+1)(\ell _2+1)}, \end{aligned}$$

and with (15) we obtain (18).

### Step 2

We now further rearrange $\overline{M}_n$ to “get rid of” the face $F^2_{d_2}$ and obtain a new EIP$_n$ minimizer which we denote $\overline{\overline{M}}_n$. To this end, recall from Definition 2.4 and Definition 2.2 that $F^1_{d_1}$ takes either the form

$$\begin{aligned} F^1_{d_1}=\mathbb {Z}^3\cap \left( ([1,a_f]\times [1,b_f]\times \{\ell _3+1\}) \cup (\{a_f+1\}\times (0,e_f]\times \{\ell _3+1\}) \right) \end{aligned}$$

or the form

$$\begin{aligned} F^1_{d_1}=\mathbb {Z}^3\cap \left( ([1,a_f]\times [1,b_f]\times \{\ell _3+1\}) \cup ((0,e_f]\times \{b_f+1\}\times \{\ell _3+1\}) \right) , \end{aligned}$$

for some $a_f\in \{1,\ldots , \ell +1\}$, $b_f\in \{a_f,a_f+1\}$, $e_f\in \{0,\ldots , b_f-1\}$, and that $F^2_{d_2}{\setminus } F^1_{d_1}$ is a rectangle $R(e,s'')$ with $e \in \{0,1,\ldots ,\ell '\}$ and $s'' \in \{1,\ldots ,\ell _3 \}$. If $e=0$ we set $\overline{\overline{M}}_n:=\overline{M}^1_n$, where

20 $$\begin{aligned} \overline{M}^1_n:=\overline{M}_n=(\mathbb {Z}^3\cap Q(\ell +1,\ell '+1,\ell _3))\cup F^1 \end{aligned}$$

for $$F^1:=F^1_{d_1}$$$$=\overline{M}_n(\cdot ,\cdot ,\ell _3+1)$$.

For $e>0$ and $F^1_{d_1}\cap F^2_{d_2}=\emptyset$ we define $\overline{\overline{M}}_n$ by distinguishing 3 cases: $a_f\ge e\vee s''$, $a_f<e$ and $a_f<s''$. Later we will see how to treat the case $F^1_{d_1}\cap F^2_{d_2}\ne \emptyset$.

1. If $a_f\ge e\vee s''$, then we move $F^2_{d_2}$ on top of $F^1_{d_1}$, and we consider the cuboidification of such configuration, which has the form
  21 $$\begin{aligned} \overline{M}^2_n:=(\mathbb {Z}^3\cap Q(\ell +1,\ell '+1,\ell _3+1))\cup F^2 \end{aligned}$$
  for some $F^2:= \overline{M}^2_n(\cdot ,\cdot ,\ell _3+2)$ (which we take to be rearranged as a daisy). We set $\overline{\overline{M}}_n:=\overline{M}^2_n$.
2. Let $a_f<e$. We can assume without loss of generality that $s''=\ell _3$. In fact, if $s''<\ell _3$, then we perform for $j=1,\dots , \ell _3 -s''$ the following transformation $\mathcal {T}^1_j$: Move an edge with length less than *e* from $F^1_{d_1}$ onto $F^2_{d_2}$, so that $F^2_{d_2}$ becomes a rectangle $R(e,s''+j)$ after removing (as done in Step (ii) of Definition 2.2) any 3-vacancy which might have been created. The obtained configuration is
  $$\begin{aligned} M^{b}_n:=(\mathbb {Z}^3\cap Q(\ell +1,\ell '+1,\ell _3 ))\cup F^{b}_1\cup F^{b}_2 \end{aligned}$$
  where $F^{b}_1:=M^b_n(\cdot ,\cdot ,\ell _3+1)$ and $F^{b}_2$ is the rectangle $R(e,\ell _3 )$. We then iterate the following transformation $\mathcal {T}^2_p$: for every element $p\in B_e$ where
  22 $$\begin{aligned} B_e:=\mathbb {Z}^3\cap (\{\ell +2\}\times (e, \ell '+1]\times \{1\}) \end{aligned}$$
  remove an edge of $F^{b}_1$, attach it to $F^{b}_2$ by first rotating it in order to make it parallel to $\varvec{e}_3$ and then translating it in such a way that one of its endpoints coincides with *p*, and then remove all 3-vacancies possibly created as in Step (ii) of Definition 2.2, so that $F^{b}_2$ becomes $R(e+1,\ell _3)$. We notice that we can perform $\mathcal {T}^2_p$ since $a_f<e\le \ell '\le \ell +1\le \ell _3+1$ by (18), thus $a_f\le \ell _3$. The configuration obtained after performing $\mathcal {T}^2_p$ for every $p\in B_e$ is
  23 $$\begin{aligned} \overline{M}^3_n:= (\mathbb {Z}^3\cap Q(\ell +2,\ell '+1,\ell _3 ))\cup F^3 \end{aligned}$$
  where $F^3:= \overline{M}^3_n(\cdot ,\cdot ,\ell _3+1)$ can be rearranged as a daisy. We set $\overline{\overline{M}}_n:=\overline{M}^3_n$.
3. Let $a_f<s''$. We first perform the transformation $\mathcal {T}^2_p$ for every $p\in B_e$ to obtain the configuration
  $$\begin{aligned} M^{c}_n:=(\mathbb {Z}^3\cap Q(\ell +1,\ell '+1,\ell _3))\cup F^c_1\cup F^c_2 \end{aligned}$$
  where $F^c_1:=M^c_n(\cdot ,\cdot ,\ell _3+1)$ and $F^c_2$ is a rectangle $R(\ell '+1,s'')$. Then, if $s''<\ell _3$ we perform $\mathcal {T}^1_j$ for every $j=1,\dots , \ell _3 -s''$ (without losing bonds since $a_f\le \ell +1\le \ell '+1$) and we obtain also a configuration of the type $\overline{M}^3_n$. Therefore, also in this case we set $\overline{\overline{M}}_n:=\overline{M}^3_n$.

Let us now consider, for $e>0$, the case $F^1_{d_1}\cap F^2_{d_2}\ne \emptyset$, which is possible according to Definition 2.2. The latter definition implies in such case $s''=\ell _3$, $a_f=\ell +1$, and

$$\begin{aligned} F^1_{d_1}\cap F^2_{d_2}=\mathbb {Z}^3\cap (\{\ell +2\}\times [1,\ldots , e_f] \times \{\ell _3+1\}) \end{aligned}$$

with $1\le e_f\le e$. Since $e\le \ell '\le \ell +1$, we have $e_f\le \ell +1$, and thanks to (18) we obtain $e_f\le \ell _3+1$. If $e_f\le \ell _3=s''$, we may move the $e_f$ points of $F^1_{d_1}\cap F^2_{d_2}$ to $\mathbb {Z}^3\cap (\{\ell +2\}\times \{e+1\}\times [1, e_f])$ and preserve the number of bonds (since $e_f\le s''$). This produces a new configuration, with the same structure of $\overline{M}_n$, but with $F^1_{d_1}\cap F^2_{d_2}=\emptyset$, and starting from such configuration we can proceed as above with the three cases. Else if $e_f=\ell _3+1$, then by $e_f\le \ell +1$ and by (18) we get $\ell =\ell _3$, hence $a_f=\ell +1=\ell _3+1=s''+1$. On the other hand, $e\le \ell '\le \ell +1=a_f\le b_f$. Therefore it is possible to remove the entire $F^2_{d_2}$ and place it above the rectangle $[1,a_f]\times [1,b_f]$ of $F^1_{d_1}$ and conclude by arguing as in Case 1 above.

We observe that $\overline{\overline{M}}_n\in \{\overline{M}^i\,:\, i=1,2,3\}$ where $\overline{M}^i$ are defined for $i=1,2,3$ in (20), (21), and (23), respectively, and hence,

24 $$\begin{aligned} \overline{\overline{M}}_n:= (\mathbb {Z}^3\cap Q(a,\ell '+1,c))\cup F_d, \end{aligned}$$

where $a\in \{\ell +1,\ell +2\}$, $c\in \{\ell _3,\ell _3+1\}$, and $F_d:=\overline{\overline{M}}_{n }(\cdot ,\cdot ,c+1)$ with $d:=\#F_d$. From here on, we assume that $a=\ell +1$. The rest of the proof for the case $a=\ell +2$ is essentially the same and we omit the details.

### Step 3

In this step we show that

25 $$\begin{aligned} \ell _3-\ell =\sqrt{6}\alpha ^{1/4}\ell ^{1/4} +\, \mathrm{o}(\ell ^{1/4}), \end{aligned}$$

where $\alpha \in [0,1)$ is specified later on in (32) and depends on $\ell$ and $\ell _3$ only.

Assume without loss of generality that $\ell _3-\ell \ge 4$. Then there exists $k\in \mathbb {N}$ such that

26 $$\begin{aligned} (c-1)-\ell =3k +r \end{aligned}$$

for some $r\in \{0,1,2\}$. We further rearrange $\overline{\overline{M}}_n$ to obtain a new minimizer $\widetilde{M}_n$ which is closer to a cube by chopping off atomic layers of thickness *k* from the top and reattaching them at the lateral boundaries. More precisely, in order to define $\widetilde{M}_n$ we consider the following subsets of $\overline{\overline{M}}_n$

27 $$\begin{aligned} S_1&:=\bigcup _{z=z_1+1,\cdots ,z_2} \overline{\overline{M}}_n(\cdot ,\cdot ,z),\nonumber \\ S_2&:=\bigcup _{z=z_2+1,\cdots ,z_3} \overline{\overline{M}}_n(\cdot ,\cdot ,z),\nonumber \\ S_3&:=F_d\cup \left( \bigcup _{z=z_3+1,\cdots ,z_4} \overline{\overline{M}}_n(\cdot ,\cdot ,z)\right) ,\nonumber \\ \quad \text {and}\quad R&:=\bigcup _{z=\ell +2,\cdots ,z_1} \overline{\overline{M}}_n(\cdot ,\cdot ,z), \end{aligned}$$

where $z_1:=\ell +1+ r$, $z_2:=\ell +1+ r+k$, $z_3:=\ell +1+ r+2k$, and $z_4:=\ell +1+ r+3k= c$. Notice that the configuration

$$\begin{aligned} G:=\overline{\overline{M}}_n{\setminus }\left[ R\cup \left( \bigcup _{k=1,2,3} S_k\right) \right] \end{aligned}$$

is such that $G(\cdot ,\cdot ,z):=R(\ell +1,\ell '+1)$ for all $1\le z\le \ell +1$ (and $G(\cdot ,\cdot ,z):=\emptyset$ for $z>\ell +1$).

We then define $\widetilde{M}_n$ as the configuration resulting by performing the following transformations:

1. Move $S_3$ altogether in such a way that each element on $\overline{\overline{M}}_n(\cdot ,\cdot ,z_3+1)$ loses its bond with $\overline{\overline{M}}_n(\cdot ,\cdot ,z_3)$ and gains a bond with
  $$\begin{aligned} G\cap \{k_1\varvec{e}_1+ \varvec{e}_2+ k_3 \varvec{e}_3\,\,:\quad k_i\in \mathbb {Z}\text { for }i=1,3\}. \end{aligned}$$
  Note that this first rearrangement does not change the total number of bonds of the minimizer $\overline{\overline{M}}_n$.
2. We then move $S_2$ altogether in such a way that each element on $\overline{\overline{M}}_n(\cdot ,\cdot ,z_2+1)$ loses its bond with $\overline{\overline{M}}_n(\cdot ,\cdot ,z_2)$ and gains a bond with
  $$\begin{aligned} G\cap \{\varvec{e}_1+ k_2\varvec{e}_2+ k_3 \varvec{e}_3\,\,:\quad k_i\in \mathbb {Z}\text { for }i=2,3\}. \end{aligned}$$
  Again, this rearrangement does not change the total number of bonds of the minimizer.
3. Observe that, after Transformation 2 and a translation of $k\varvec{e}_1+k\varvec{e}_2$ the resulting configuration, which we denote by $T_n$, is contained in the cuboid $[1,\ell +1+k]\times [0,\ell '+1+k]\times [1,\ell +1+r+k]$, (0 in the second factor is due to the new placement of $F_d$ after moving $S_3$). However, $T_n$ does not contain the points of $V:=\mathbb {Z}^3\cap (V_1\cup V_2\cup V_3)$, where
  $$\begin{aligned} \begin{aligned} V_1&:=[k+1,\ell +1+k]\times [1,k]\times [\ell +2,\ell +r+k] \\ V_2&:= [1,k]\times [k+1, \ell +k+1]\times [\ell +2,\ell +r+k]\\ V_3&= [1,k]\times [1,k]\times [1,\ell +1]. \end{aligned} \end{aligned}$$
  Notice that $V_1=V_2=\emptyset$ if $k=1$ and $r=0$. We now fill-in the set *V* by subsequently moving edges with length $\ell$ from the side aligned in the direction $\varvec{e}_1$ of $T_n (\cdot ,\cdot ,z_2)$ (each contains $\ell +1$ points). We call the resulting configuration $\widetilde{M}_n$ and we denote its (remaining) upper face $\widetilde{M}_n(\cdot ,\cdot ,z_2)$ by $\widetilde{F}_m$ with
  28 $$\begin{aligned} \begin{aligned} m:=\#\widetilde{F}_m&=(\ell +1)(\ell '+1)-\#V\\&=(\ell +1)(\ell '+1)-(\ell +1)[k^2+2k(r+k-1)]. \end{aligned} \end{aligned}$$
  Notice that in all the steps the total number of bonds of the configuration remains the same as in $M_n$, and so along these transformations the edges with length $\ell$ are not exhausted before *V* is filled (since this would contradict minimality of $\overline{\overline{M}}_n$ for the EIP$_n$). Hence, $\widetilde{M}_n$ is an EIP$_n$ minimizer as well.

By (28) we have

29 $$\begin{aligned} m&=(\ell +1)(\ell '+1)-\#V=(\ell +1)(\ell '+1)-(\ell +1)\left[ k^2\,+\,2k(k+r-1) \right] \nonumber \\&=(\ell +1)(\ell '+1-(k^2+2k(k+r-1))) \nonumber \\&=(\ell +1)(\ell -3k^2-2s_1k+s_2+1)\nonumber \\&=\ell ^2 -\ell (3k^2+2s_1k-s_2-2) -3k^2-2s_1k+s_2+1 \end{aligned}$$

where $$s_1:=r-1\in \{-1,0,1\}$$ and $$s_2:=\ell '-\ell \in \{0,1\}$$.

Furthermore, as $\widetilde{M}_n$ is a minimizer of the EIP$_n$, it is not possible to gain any bond by rearranging the elements of $\widetilde{F}_m$ over $\widetilde{M}_n{\setminus }\widetilde{F}_m$. Therefore, $\widetilde{F}_m$ is a minimizer (up to translation) of EIP$^2_m$ (see (7)). By (9),

30 $$\begin{aligned} \Theta _{2}(\widetilde{F}_m) = 2\lceil 2\sqrt{m} \rceil . \end{aligned}$$

Since by the Transformation 3 the configuration $\widetilde{F}_m$ is rectangular with side lengths $\ell$ and $\ell '-\left( k^2\,+\,2k(k+r-1)\right)$ its edge perimeter is simply

31 $$\begin{aligned} \Theta _{2}(\widetilde{F}_m)&= 2(\ell + 1) +2(\ell '-k^2-2k(k+r-1)+1) \nonumber \\&=4\ell -6k^2-4s_1k +2s_2 + 4. \end{aligned}$$

Therefore, by (29), (30), and (31), we have that

$$\begin{aligned}&4\ell -6k^2-4s_1k +2s_2\\&\quad = 2\lceil 2\sqrt{\ell ^2 -\ell (3k^2+2s_1k-s_2-2) -3k^2-2s_1k+s_2+1} \rceil - 4, \end{aligned}$$

which can be written as

$$\begin{aligned}&2\ell -3k^2-2s_1k +s_2\\&\quad = 2\sqrt{\ell ^2 -\ell (3k^2+2s_1k-s_2-2) -3k^2-2s_1k+s_2+1} - 2 +\alpha \end{aligned}$$

with

32 $$\begin{aligned} \alpha :=\lceil 2\sqrt{m} - 1\rceil \,-\,( 2\sqrt{m} - 1)=\lceil 2\sqrt{m}\rceil \,-\,2\sqrt{m}\in [0,1). \end{aligned}$$

By taking the square we obtain

$$\begin{aligned}&(2\ell -3k^2-2s_1k +s_2 + 2-\alpha )^2\\&\quad =4\ell ^2 -4\ell (3k^2+2s_1k-s_2-2) -12k^2-8s_1k+4s_2+4 \end{aligned}$$

from which it is straightforward to compute

33 $$\begin{aligned} 4\alpha \ell =9k^4 +12s_1k^3 +2(2s_1^2 + 3\alpha -3s_2)k^2 +4s_1(\alpha -s_2)k + (\alpha -s_2)^2-4\alpha . \end{aligned}$$

We now observe that (33) yields

34 $$\begin{aligned} k=\frac{\sqrt{2}\,\alpha ^{1/4}}{\sqrt{3}} \ell ^{1/4} +\, \mathrm{o}(\ell ^{1/4}). \end{aligned}$$

Therefore, from (26) and (34) we obtain

35 $$\begin{aligned} \ell _3-\ell =\sqrt{6}\alpha ^{1/4}\ell ^{1/4} +\, \mathrm{o}(\ell ^{1/4}). \end{aligned}$$

### Step 4

In this step we show that (with $\ell _n$ as defined in (3))

36 $$\begin{aligned} \ell _n-\ell = \sqrt{\frac{2}{3}}\, \alpha ^{1/4}\ell ^{1/4} + \,\mathrm{o} (\ell ^{1/4}). \end{aligned}$$

From (24) we have that

37 $$\begin{aligned} n&=(\ell +1)\, (\ell '+1) \, (\ell _3 + s_3) \,+\, d = (\ell +1) \,(\ell '+1) \,(\ell +\ell _3-\ell +s_3 ) \,+\,d \nonumber \\&= \ell ^3 \,+\, (\ell _3-\ell )\, \ell ^2 \, +\,\mathrm{O} (\ell ^2) \end{aligned}$$

where $s_2:=\ell '-\ell \in \{0,1\}$ and $s_3 = c - \ell _3$, since $d=\mathrm{O}(\ell ^2)$ and since $\ell _3-\ell =\mathrm{O}(\ell ^{1/4})$ by (35). Then, (37) together with (3) yields

38 $$\begin{aligned} \ell _n&=\lfloor \root 3 \of {n}\rfloor =\left\lfloor \ell \,\root 3 \of {1\,+\,\frac{\ell _3-\ell }{\ell }\, +\, \mathrm{O} \left( \frac{1}{\ell }\right) }\right\rfloor \nonumber \\&= \left\lfloor \ell \,\root 3 \of {1\,+\,\sqrt{6}\,\alpha ^{1/4}\ell ^{-3/4} + \,\mathrm{o} (\ell ^{-3/4})}\right\rfloor \nonumber \\&= \left\lfloor \ell \,\left( 1 +\frac{\sqrt{6}\alpha ^{1/4}}{3} \ell ^{-3/4} + \,\mathrm{o} (\ell ^{-3/4})\right) \right\rfloor , \end{aligned}$$

where in the second equality we used (35). The assertion (36) follows now from (38), since it implies

39 $$\begin{aligned} \ell _n-\ell =\left\lfloor \sqrt{\frac{2}{3}}\, \alpha ^{1/4}\ell ^{1/4} + \,\mathrm{o} (\ell ^{1/4})\right\rfloor . \end{aligned}$$

### Step 5

In this step we conclude the proof of the estimate (16).

Let us define $\varepsilon _i\in \mathbb {R}$ such that $\ell _i=\ell (1+\varepsilon _i)$. We begin by observing that, as a consequence of Step 4 we have that

40 $$\begin{aligned} \ell =n^{1/3}+\mathrm{o}(n^{1/3}). \end{aligned}$$

Furthermore, from (35) it follows that

41 $$\begin{aligned} \varepsilon _3 = \frac{\ell _3-\ell }{\ell } \le \sqrt{6}\,\alpha ^{1/4}\ell ^{-3/4} + \,\mathrm{o} (\ell ^{-3/4}). \end{aligned}$$

By (15) and (19) we have $\ell _2\ge \ell$. Therefore, by (41) we obtain that

42 $$\begin{aligned} 0\le \varepsilon _2\le \varepsilon _3\le \sqrt{6}\,\alpha ^{1/4}\ell ^{-3/4} + \,\mathrm{o} (\ell ^{-3/4}) \end{aligned}$$

as $\ell _2\le \ell _3$. If also $\ell _1\ge \ell$, then the same reasoning yields that $0\le \varepsilon _1\le \varepsilon _3\le \sqrt{6}\,\alpha ^{1/4}\ell ^{-3/4} + \,\mathrm{o} (\ell ^{-3/4})$. Therefore, it only remains to consider the case in which $\ell _1<\ell$ and hence, $\varepsilon _1<0$. We have in such case, again by (15) and (19),

$$\begin{aligned}&\ell ^2\le \ell _1 \ell _2 \ \Rightarrow \ \ell ^2 \le \ell ^2 (1+\varepsilon _1)(1+\varepsilon _2) \ \Rightarrow \ 0 \le \varepsilon _1+\varepsilon _2+\varepsilon _1\varepsilon _2 \nonumber \\&\quad \Rightarrow \ -\varepsilon _1 \le \varepsilon _2 + \varepsilon _1\varepsilon _2 \end{aligned}$$

so that, in particular

43 $$\begin{aligned} 0\le -\varepsilon _1 \le \varepsilon _2. \end{aligned}$$

Therefore, by (41), (42), and (43) we conclude that

44 $$\begin{aligned} |\varepsilon _i|\le \sqrt{6}\,\alpha ^{1/4}\ell ^{-3/4} + \,\mathrm{o} (\ell ^{-3/4}). \end{aligned}$$

for $i=1,2,3$. Finally, by Step 4 and (44) we observe that

$$\begin{aligned} |\ell _i-\ell _n|&\le |\ell _i-\ell |+|\ell -\ell _n|\nonumber \\&\le \ell |\varepsilon _i|+ \sqrt{\frac{2}{3}}\, \alpha ^{1/4}\ell ^{1/4} + \,\mathrm{o} (\ell ^{1/4})\nonumber \\&\le \left( \sqrt{6}+ \sqrt{\frac{2}{3}}\right) \alpha ^{1/4}\ell ^{1/4} + \,\mathrm{o} (\ell ^{1/4}) \end{aligned}$$

for $i=1,2,3$, which in turn by (40) yields estimate (16) with

$$\begin{aligned} K:= \left( \sqrt{6}+ \sqrt{\frac{2}{3}}\right) \alpha ^{1/4} \end{aligned}$$

where we recall that $\alpha :=\lceil 2\sqrt{m}\rceil \,-\,2\sqrt{m}\in [0,1)$, see (32), for *m* given by (28). $\square$

## Lower Bound: Proof of Theorem 1.2

We begin this section with an auxiliary lemma about solutions to the EIP in the two-dimensional square lattice. Indeed, for nonnegative integers *s*, *p*, *q* (with $s>p\vee q)$, we consider configurations in $\mathbb {Z}^2$ of the form

$$\begin{aligned} \mathcal {R}_{s,p,q}:= R(s-p-1,s)\cup L^p_{s-q}, \end{aligned}$$

where $L^p_{s-q}:=\mathbb {Z}^2\cap (\{s-p\}\times [1, s-q])$. Note that $\#\mathcal {R}_{s,p,q}=s^2-sp-q$.

### Lemma 4.1

Let $s,p,q\in \mathbb {N}\cup \{0\}$ be such that $s\ge 1$, $p< s$, $q<s$. Then $\mathcal {R}_{s,p,q}$ is an EIP$_n^2$ minimizer (where $n=\#\mathcal {R}_{s,p,q}=s^2-sp-q$) if and only if

$$\begin{aligned} 4(s-q)>(p+1)^2. \end{aligned}$$

In particular, by choosing $p=\lfloor s^{1/2}\rfloor$ and $q=\lfloor s/4\rfloor$, $\mathcal {R}_{s,p,q}$ is a EIP$_n^2$ minimizer for any $s\ge 2$.

### Proof

We observe that the number of unit bonds in $\mathcal {R}_{s,p,q}$ is equal to

$$\begin{aligned} (s-1)(s-p-1)+s(s-p-2)+2(s-q)-1. \end{aligned}$$

We use the fact that EIP$_n^2$ minimizers are characterized by a number of unit bonds equal to $\lfloor 2n-2\sqrt{n}\rfloor$, as recalled in Sect. 2. As a consequence, $\mathcal {R}_{s,p,q}$ is an EIP$_n^2$ minimizer if and only if

$$\begin{aligned} (s-1)(s-p-1)+s(s-p-2)+2(s-q)-1=\lfloor 2(s^2-sp-q)-2\sqrt{s^2-sp-q}\rfloor , \end{aligned}$$

which is equivalent to $\lfloor 2s-p-2\sqrt{s^2-sp-q} \rfloor =0$, thus to

$$\begin{aligned} 0\le 2s-p-2\sqrt{s^2-sp-q} <1. \end{aligned}$$

As the first inequality is obvious, $\mathcal {R}_{s,p,q}$ is an EIP$_n^2$ minimizer if and only if

$$\begin{aligned} 2s-p-1< 2\sqrt{s^2-sp-q}, \end{aligned}$$

which is equivalent to $4(s-q)>(p+1)^2$, as desired.

By choosing $p=\lfloor s^{1/2}\rfloor$ and $q=\lfloor s/4\rfloor$, the latter is reduced to

$$\begin{aligned} 4s>1+2\lfloor \sqrt{s}\rfloor +4\lfloor s/4\rfloor +\lfloor \sqrt{s}\rfloor ^2, \end{aligned}$$

which is implied by $2s>1+2\sqrt{s}$ that is clearly true for $s\ge 2$. $\square$

A straightforward consequence of Lemma 4.1 is the sharpness of the $N^{3/4}$ law in the two-dimensional square lattice, see [17]. Indeed, we might consider the sequence

45 $$\begin{aligned} d_s:=s^2-s\lfloor s^{1/2}\rfloor -\lfloor s/4 \rfloor ,\qquad s=2,3,\ldots . \end{aligned}$$

It is easy to check that $d_s$ is a strictly increasing sequence. We have $d_s=\#\mathcal {R}_{s,p,q}$ with $p=\lfloor s^{1/2}\rfloor$ and $q=\lfloor s/4\rfloor$, and $\mathcal {R}_{s,p,q}$ is an EIP$_{d_s}^2$ minimizer by Lemma 4.1. On the other hand,

$$\begin{aligned} s-\lfloor d_s^{1/2}\rfloor = \left\lceil s-s\sqrt{1-\frac{sp+q}{s^2}} \right\rceil \ge s-s\left( 1-\frac{sp+q}{2s^2}\right) \ge \frac{\lfloor s^{1/2}\rfloor }{2}, \end{aligned}$$

so that we may compare the two-dimensional Wulff shape $W_{d_s}^2:=\big [1, \lfloor d_s^{1/2} \rfloor \big ]^2\cap \mathbb {Z}^2$ with $\mathcal {R}_{s,p,q}$ and get

$$\begin{aligned} \min _{a\in \mathbb {Z}^2}\#\Big (\mathcal {R}_{s,p,q}\,\triangle \, \Big (a+ W_{d_s}^2\Big )\Big ) \ge \frac{1}{2} \lfloor s^{1/2}\rfloor \left( s-\lfloor s^{1/2}\rfloor -1\right) , \end{aligned}$$

for any $s\ge 2$. As (45) implies $s=d_s^{1/2}+ \mathrm{o} (d_s^{1/2})$, we find

$$\begin{aligned} \min _{a\in \mathbb {Z}^2}\#\big (\mathcal {R}_{s,p,q}\,\triangle \, \big (a+ W_{d_s}^2\big )\big )\ge \frac{1}{2} d_s^{3/4}+ \mathrm{o} \big (d_s^{3/4}\big ). \end{aligned}$$

We now proceed with the proof of the three-dimensional counterpart of this result.

### Proof of Theorem 1.2

Let us consider the strictly increasing sequence

$$\begin{aligned} n_s:=s^3+s^2-s\lfloor s^{1/2}\rfloor -\lfloor s/4 \rfloor ,\quad s=2,3,\ldots . \end{aligned}$$

For any integer $s\ge 2$, we may consider the configuration

46 $$\begin{aligned} M_{n_s}:=\left( Q(s,s,s)\cap \mathbb {Z}^3\right) \cup F_{d_s} \end{aligned}$$

where we have introduced a 2-dimensional configuration $F_{d_s}:=M_{n_s}(\cdot ,\cdot ,s+1)$ with $d_s:=\#M_n(\cdot ,\cdot ,s+1)$. More precisely, we define the top face $F_{d_s}$ as

47 $$\begin{aligned} F_{d_s}:=\left( \mathbb {Z}^3\cap \left( [ 1,r]\times [1,s]\times \{s+1\}\right) \right) \cup L^{s},\quad r:=s-\lfloor s^{1/2}\rfloor -1, \end{aligned}$$

where

48 $$\begin{aligned} L^{s}:=\mathbb {Z}^3\cap (\{r+1\}\times [1,s-q]\times \{s+1\}), \quad q:=\lfloor s/4\rfloor . \end{aligned}$$

We see that

49 $$\begin{aligned} d_s=s^2-s\lfloor s^{1/2}\rfloor -\lfloor s/4 \rfloor <s^2 \end{aligned}$$

and that

50 $$\begin{aligned} n_s=s^3+d_s \end{aligned}$$

is indeed the number of points of $M_{n_s}$. Moreover, the top face $F_{d_s}$ is an EIP$^2_{d_s}$ minimizer for any $s\ge 2$ by an application of Lemma 4.1. This ensures minimality of $M_{n_s}$ for the EIP$_{n_s}$, for any $s\ge 2$, cf. [6]. We stress that

51 $$\begin{aligned} s-r=\lfloor s^{1/2}\rfloor +1. \end{aligned}$$

By (49) and (50) it follows that

52 $$\begin{aligned} s=n_s^{1/3} + \mathrm{o}(n_s^{1/3}), \end{aligned}$$

and hence

53 $$\begin{aligned} r=n_s^{1/3} + \mathrm{o}\big (n_s^{1/3}\big ) \end{aligned}$$

by (51). Furthermore, by (49) and (52) we have

54 $$\begin{aligned} d_s=n_s^{2/3} + \mathrm{o}\big (n_s^{2/3}\big ). \end{aligned}$$

We also refine (52) by recalling (3) and by claiming that

55 $$\begin{aligned} s=\ell _{n_s}. \end{aligned}$$

To prove (55) we observe that $s\le \ell _{n_s}$ easily follows from (50) and that

56 $$\begin{aligned} \ell _{n_s}&=\lfloor \root 3 \of {n_s-d_s+d_s}\rfloor =\left\lfloor \root 3 \of {n_s-d_s}\,\root 3 \of {1+\frac{d_s}{n_s-d_s}}\right\rfloor \nonumber \\&=\left\lfloor s\,\root 3 \of {1+\frac{d_s}{s^3}}\right\rfloor \le \left\lfloor s\,\Big (1+\frac{1}{3}\frac{d_s}{s^3}\Big ) \right\rfloor =s \end{aligned}$$

where we used (3) in the first equality, (50) in the third one, (49) in the last equality. The claim is proved.

**Fig. 5:**
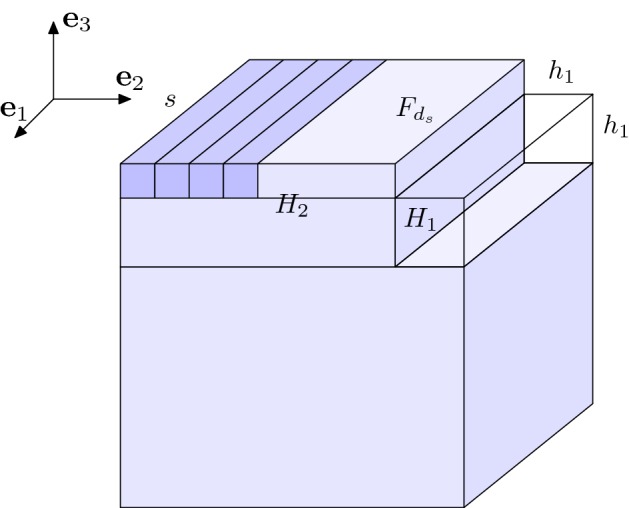
Configuration $$M'_{n_s}$$

We then proceed to construct another minimizer denoted by $M''_{n_s}$ by performing the following two consecutive transformations on $M_{n_s}$:

1. Define the integer
  57 $$\begin{aligned} h_1=\left\lfloor \frac{1}{3}n_s^{1/12}\right\rfloor . \end{aligned}$$
  We translate by $\varvec{e}_1$ the top face $F_{d_s}$ and we move altogether the edge $\left( \{s\}\times [1,s] \times \{s\}\right) \cap \mathbb {Z}^3$ to the position $\left( \{1\}\times [1,s]\times \{s+1\}\right) \cap \mathbb {Z}^3$. We repeat then this procedure recursively for each line (parallel to $\varvec{e}_2$) with *s* elements of $M_{n_s}$ which is included in the set
  $$\begin{aligned} H_1:=[s-h_1,s]\times [1,s]\times [s-h_1,s]. \end{aligned}$$
  This transformation gives the configuration $$M'_{n_s}$$, see Fig. 5. Note that $$M'_{n_s}$$ is an EIP$$_{n_s}$$ minimizer for large enough *s*. In fact, the total number of moved lines is $$(h_1+1)^2$$. Hence, we can translate $$F_{d_s}$$ by $$(h_1+1)^2\varvec{e}_1$$ without losing any bond if $$ r+1+(h_1+1)^2<s-h_1$$, as such a condition prevents the top face from reaching the points above the ‘hole’ $$H_1$$ by this translation. The latter inequality is equivalent, by (51), to
  58 $$\begin{aligned} h_1+(h_1+1)^2<\lfloor s^{1/2}\rfloor , \end{aligned}$$
  which holds true for large enough *s* due to (55) and since by definition of $h_1$ we have
  $$\begin{aligned} (h_1+1)^2=\frac{1}{9}n_s^{1/6} + \mathrm{o}(n_s^{1/6}). \end{aligned}$$
2. Thanks to the previous step, there exists $s_0\in \mathbb {N}$ such that, for any $s\ge s_0$, $M'_{n_s}$ is an EIP$_{n_s}$ minimizer. In particular, $s_0$ can be defined as the smallest integer such that (58) hold for any $s\ge s_0$. For $s\ge s_0$ we move altogether the elements in the set
  $$\begin{aligned} H_2:=[1, s-h_1-1]\times [1,s]\times [s-h_1,s+1] \end{aligned}$$
  in such a way that each element of $M'_{n_s}(\cdot ,\cdot ,s-h_1)$ (loses the bond with $M'_{n_s}(\cdot ,\cdot ,s-h_1-1)$ and) gets bonded with an element of $M'_n(1,\cdot ,\cdot ) {\setminus } H_2$. We denote the resulting EIP$_{n_s}$ minimizer by $M''_{n_s}$.

Thanks to the two steps above, for any $s\ge s_0$ the constructed configuration $M''_{n_s}$ is a minimizer of the EIP$_{n_s}$ problem and moreover we notice that

59 $$\begin{aligned} \mathbb {Z}^3\cap ([-h_1,s]\times [1,s]\times [1, s-h_1-1])\subset M''_{n_s}, \end{aligned}$$

therefore by (3), (55), (57), (59) we conclude that

$$\begin{aligned} \begin{aligned} \min _{a\in \mathbb {Z}^3}\#(M_{n_s}''\triangle (a+ W_{n_s}))&\ge \min _{a\in \mathbb {Z}^3}\#(M_{n_s}''{\setminus } (a+ W_{n_s}))\\ {}&\ge s(s-h_1-1)(s+h_1+1-\ell _{n_s})\\ {}&=s \left( s-\left\lfloor \frac{1}{3} \, n_s^{1/12}\right\rfloor -1\right) \, \left( \left\lfloor \frac{1}{3} n_s^{1/12}\right\rfloor +1\right) . \end{aligned} \end{aligned}$$

Hence,

$$\begin{aligned} \min _{a\in \mathbb {Z}^3}\#\big (M''_{n_s}\triangle \big (a+ W_{{n_s}}\big )\big )\ge \frac{1}{3} n_s^{3/4}+ \mathrm{o}\big (n_s^{3/4}\big ) \end{aligned}$$

follows by (52). $$\square $$

**Fig. 6:**
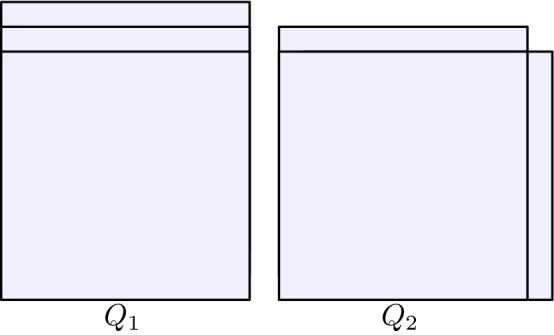
The exponent 3 / 4 is not optimal for $d\ge 5$: configurations $Q_1$ and $Q_2$

Before closing this discussion, let us point out that the exponent 3 / 4, which is proved to be optimal in $\mathbb {Z}^3$ (this paper) and in $\mathbb {Z}^2$ [17], is not optimal in $\mathbb {Z}^d$ for $d> 4$. Indeed, as *d* grows minimizers can differ by a larger portion of elements: A hypercube with two extra hyperfaces in one direction $Q_1:=[1,s]^{d-1}\times [1,s+2]$ and a hypercube with two extra hyperfaces in two different directions $Q_2:=[1,s]^{d-1}\times [1,s+1] \cup [1,s+1]\times [1,s]^{d-1}$, see Fig. 6, are both minimizers and fulfill $\#(Q_1 \triangle Q_2) = 2 s^{d-1}$. Since $s\sim n^{1/d}$ we have that $\#(Q_1 \triangle Q_2) \sim n^{(d-1)/d}$, and $(d-1)/d$ is strictly larger than 3 / 4 for all $d\ge 5$.

This argument does not allow to conclude the nonoptimality of the exponent 3 / 4 for $$d=4$$. On the other hand, we expect that the construction from Fig. 5 could be adapted to show that such exponent is nonoptimal for $$ d=4$$ as well.
